# Case Report: Diagnosis of Acute Q Fever With Aseptic Meningitis in a Patient by Using Metagenomic Next-Generation Sequencing

**DOI:** 10.3389/fmed.2022.855020

**Published:** 2022-05-18

**Authors:** Meifeng Gu, Xiaoqin Mo, Zhenchu Tang, Jianguang Tang, Wei Wang

**Affiliations:** ^1^Department of Neurology, the Second Xiangya Hospital, Central South University, Changsha, China; ^2^Hunan Key Laboratory of Tumor Models and Individualized Medicine, the Second Xiangya Hospital, Central South University, Changsha, China

**Keywords:** Q fever, Coxiella burnetii, febrile illness, neurological complications, metagenomic next-generation sequencing (mNGS), diagnostic technique

## Abstract

Query fever (Q fever) is a widespread zoonotic disease caused by the bacterium of *Coxiella burnetii* (*C. burnetii*). Its neurological complications are rarely reported. But they may lead to severe consequences. It needs a rapid and accurate detective method to diagnose acute Q fever with neurological presentations in non-epidemic areas urgently. Here, we report an acute Q fever case with aseptic meningitis. The male patient, without any contact history in the epidemic area or with animals, was indicated to exhibit fever and headache symptoms. The cultures of blood, stool, urine, and sputum were all negative. But *C. burnetii* was repeatedly detected in blood by metagenomic next-generation sequencing (mNGS). He received Doxycycline therapy and quickly returned to normal. Therefore, for the diagnosis and identification of Q fever in non-reporting regions, mNGS has comparative advantages. Secondly, aseptic meningitis may be a direct infection of *C. burnetii* to central nervous system (CNS) or inflammatory reactions to systemic infection, we recommend detecting mNGS both in blood and cerebrospinal fluid (CSF).

## Introduction

Query fever (Q fever) is a widespread zoonotic disease caused by the bacterium known as *Coxiella burnetii* (*C. burnetii*). Transmission is considered to happen *via* respiratory droplets, contact with ingestion of unpasteurized dairy products, and secretions from the primary reservoirs of infection such as cattle, sheep, goats as well as pets (1, 2). Q fever was originally identified as a rare and regionally restricted disease, but now it has a worldwide distribution (3). Although seroprevalence data are available for most countries, it can be considered that the true incidence of Q fever in humans is largely underestimated (4).

Patients who get acute Q fever commonly present with febrile illness, pneumonia, hepatitis, and severe conditions with neurological syndrome—headache and meningitis/meningoencephalitis (1, 5). For people who live in epidemic areas with common clinical manifestations, acute Q fever can be taken into consideration easily. But approximately 60% remain asymptomatic when infected with *C. burnetii* (6, 7). Because of its non-specific clinical manifestations, the diagnosis of Q fever is very difficult, especially in non-epidemic areas (8).

For the diagnosis of Q fever, serology and/or polymerase chain reaction (PCR) is the gold standard method. Physicians will test all unexplained fever patients in the epidemic areas, but in the non-epidemic areas, testing is seldom done or could not do. Fortunately, as a revolutionary diagnostic tool, metagenomic next-generation sequencing (mNGS) can be used as an early diagnostic method for all potential pathogens especially rare pathogens (including *C. burnetii*). Here, we report an acute Q fever patient presented as aseptic meningitis diagnosed by mNGS in Changsha where Q fever has never been reported before.

## Case Presentation

**Figure 1:**
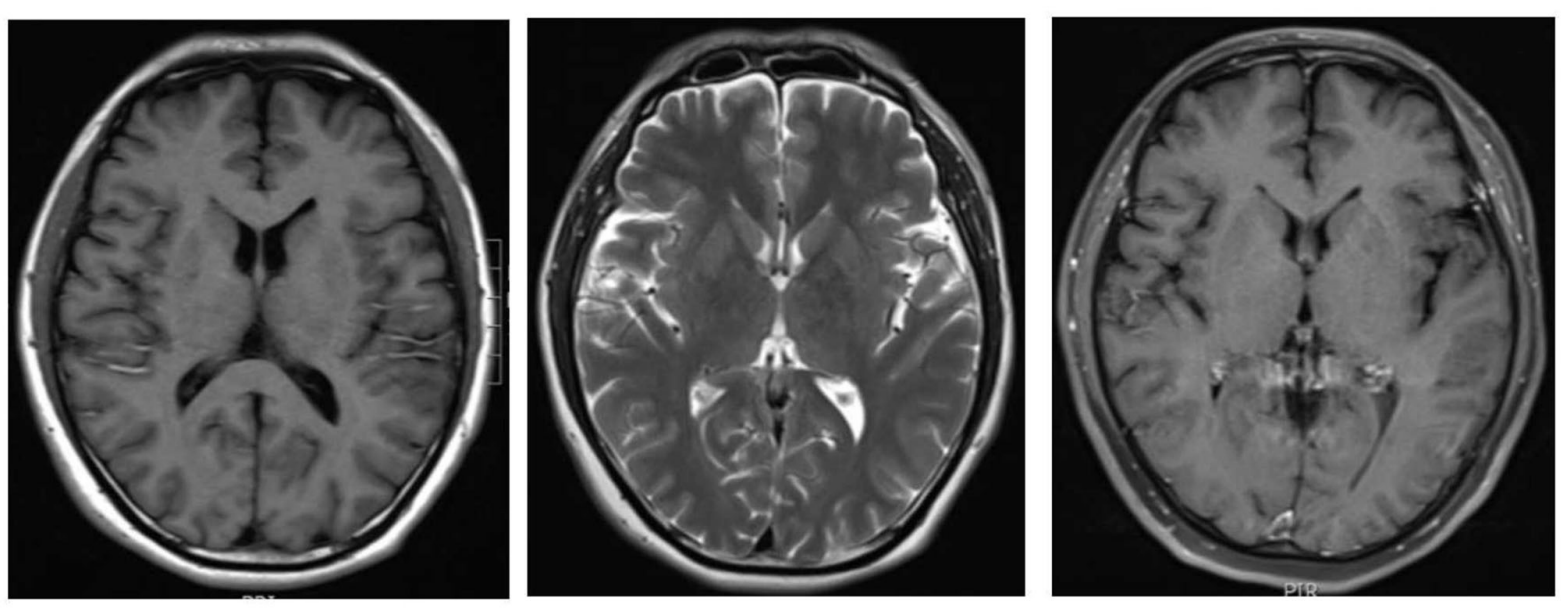
Brain magnetic resonance imaging done on the first hospital day. T1WI/T2WI/Post-Contrast T1WI didn't show any abnormality.

A previously healthy 51 years old male suffering from flu-like symptoms such as sneezing, a runny nose, fatigue and myalgia for 7 days, followed by high fever and severe headache for 4 days, was admitted to our hospital on February 4th, 2021. Physical examinations revealed only positive Kernig signs. Laboratory tests showed that hemoglobin was 103 g/l, glutamic-pyruvic transaminase 118 u/l, and aspartate aminotransferase 72 u/l but kidney function was normal. Systematic inflammatory markers were elevated, including erythrocyte sedimentation rate (36 mm/h), C reactive protein (44 mg/ml), and procalcitonin (0.98 ng/ml). Serological tests for Cytomegalovirus (CMV), herpes simplex virus I, II (HSV I, II), Epstein-Barr Virus (EBV), Widal reaction, hepatitis viruses, human immunodeficiency virus (HIV), syphilis (RPR), and tumor markers were performed and proved to be negative. Cerebrospinal fluid (CSF) examination showed moderate lymphocytic pleocytosis (25x10∧6/L white blood cell, 75% lymphocytes), normal glucose and protein levels. Brain MRI (Figure 1), lung CT, and Doppler echocardiography showed no abnormalities. Viral meningitis was made for the primary diagnosis. But we could not exclude completely the possibility of bacterial infection. Then the patient received empirical antiviral drugs (Acyclovir 500 mg/8 h intravenous drip) and antibiotics (Moxifloxacin 400 mg/day intravenous drip). Before starting empirical treatment, mNGS and conventional methods both in blood and CSF were detected.

**Figure 2:**
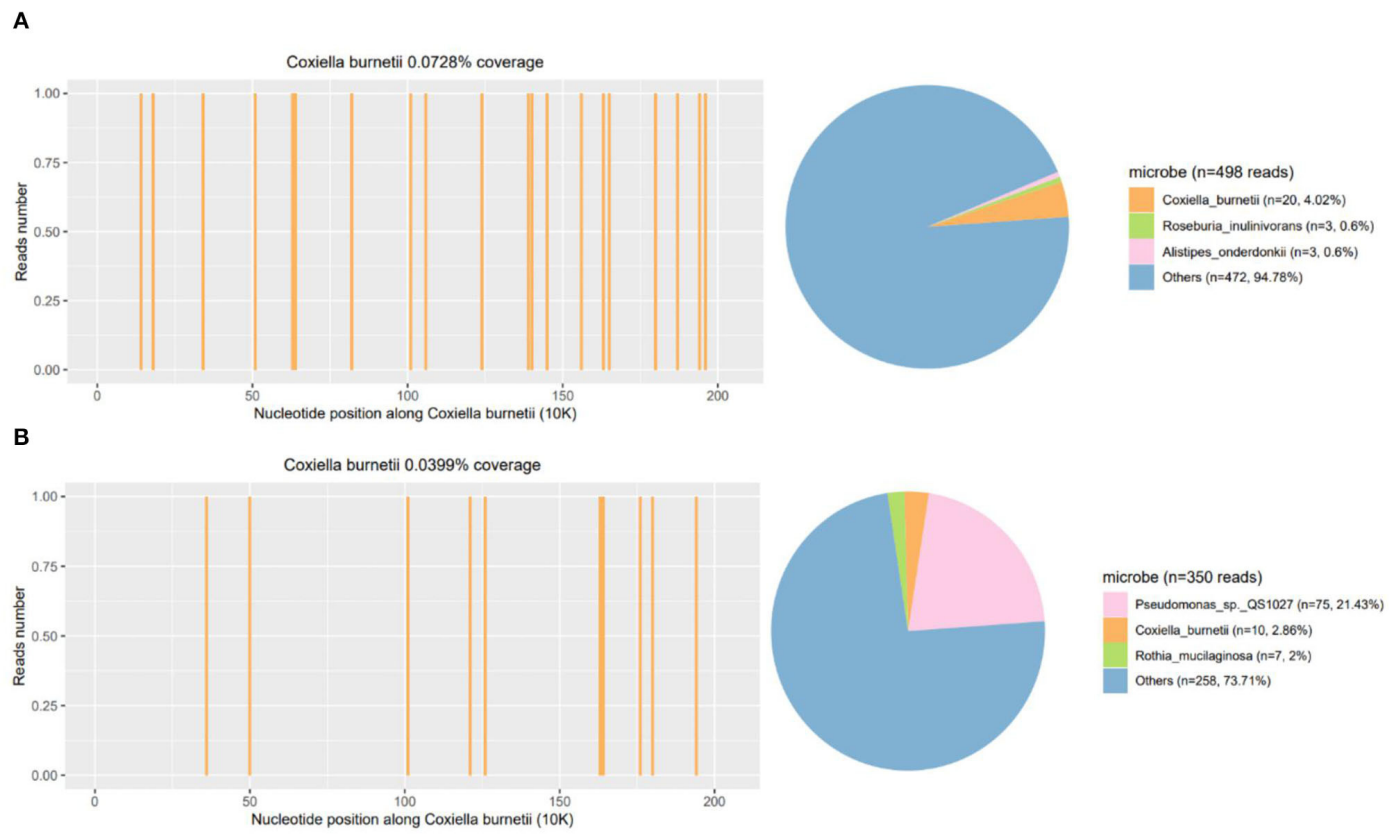
Repeated mNGS of blood results of the patient. The coverage and proportion of *Coxiella burnetii* detected by mNGS in blood **(A)** and reexamined mNGS in blood **(B)**. mNGS, metagenomics next-generation sequencing.

**Figure 3:**
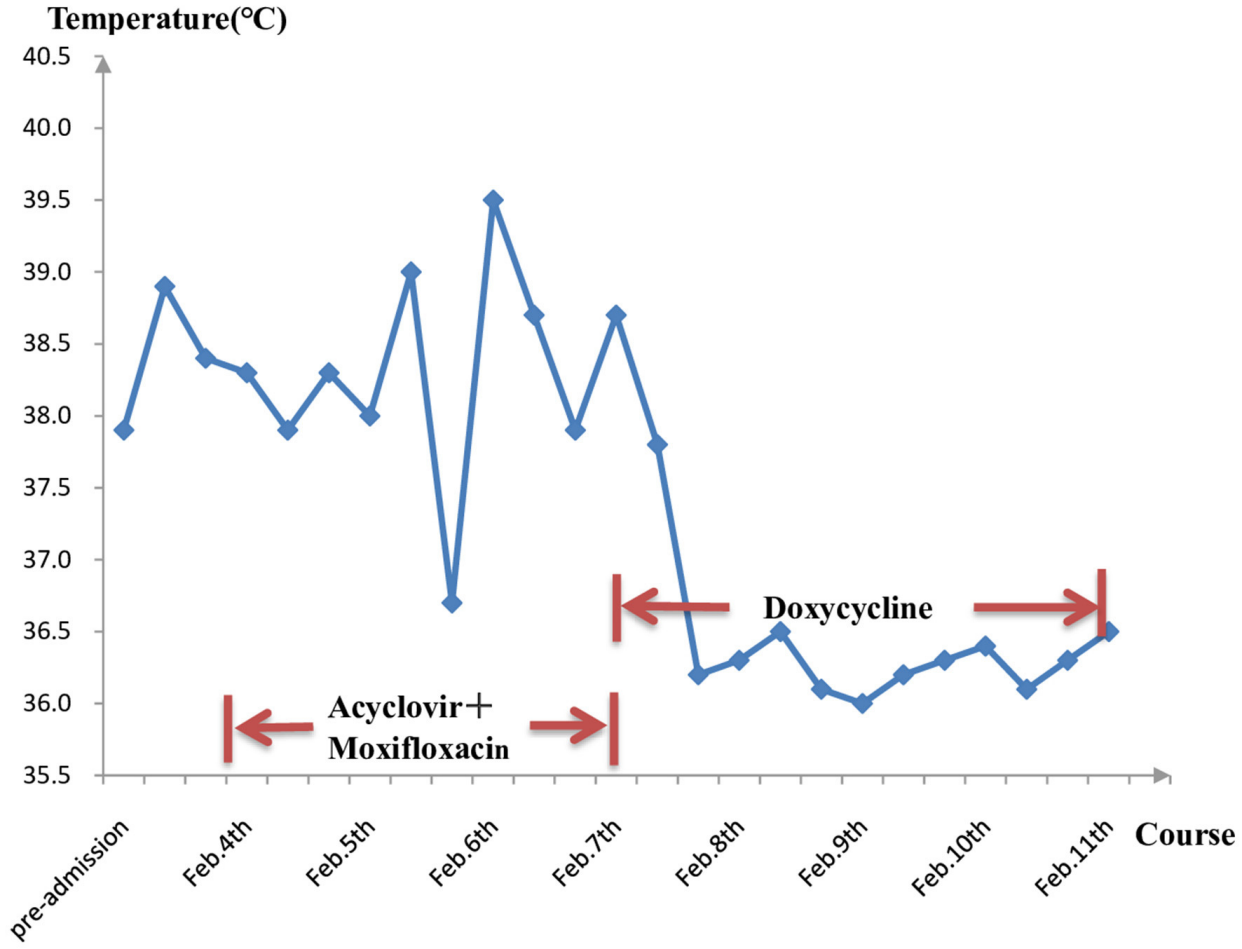
Longitudinal analysis of temperature and medication. After using specific drugs—doxycycline, the temperature was under control and didn't bounce back again.

He responded slightly to these treatments, but still had mild to moderate fever. The cultures of blood, stool, urine, and sputum were negative for pathogens. Blood mNGS detected 20 reads of *C. burnetii* on day 3 (Figure 2A) but CSF mNGS turned to a negative result. However, Q fever is uncommon in our area and the patient didn't have a history of close contact with domestic animals and Q fever patients. Serum was negative for IgM antibodies against *C. burnetii*. Therefore, blood mNGS was reexamined before adding doxycycline (100 mg twice daily) to adjustments of treatment. After the use of doxycycline, his temperature and procalcitonin returned to normal. Therefore, the diagnosis of acute Q fever had been proven by a positive of the second mNGS analysis of blood [10 unique reads of *C. burnetii* (Figure 2B)] and a good response to doxycycline. Empiric acyclovir and moxifloxacin had been stopped due to the definitive diagnosis (Figure 3). Soon he didn't have a fever anymore, meanwhile, the hemoglobin and hepatic function returned to normal. He was discharged from the hospital only with myalgias and fatigue. And during the subsequent visit, the laboratory tests including blood routine, liver function, examinations for rheumatism, and heart Doppler were all normal and now he has completely recovered.

## Discussion

It was until 1958 that Q fever was first reported in China through the serological method of the complement fixation test (CFT). Later, Q fever was reported from Tibet, Inner Mongolia, Sichuan, Zhejiang, Guangdong, Shandong, and other 20 provinces of China with an exception of Hunan province. In humans, ~60% remain asymptomatic when infected. Acute Q fever which has an incubation period of 1–3 weeks (9), usually presents as a self-limited febrile illness with flu-like manifestations, but severe conditions such as pneumonia, hepatitis, and central nervous system involvement also occur. The diagnostic criteria of acute Q fever from the U.S. Department of Centers for Disease Control and Prevention must meet the following condition (7): (1) clinical manifestations of fever and at least one of the following: rigors, severe retrobulbar headache, acute hepatitis, pneumonia, or elevated liver enzymes; (2) laboratory confirmed criteria at least one of the following: fourfold change in IgG antibody titer to *C. burnetii* phase II antigen by IFA between paired sera; detection of *C. burnetii* by PCR or IHC or culture; (3) laboratory supportive criteria: single IgG titer ≥1:128 to *C. burnetii* phase II antigen by IFA (phase I titers may be elevated as well) or elevated phase II IgG or IgM antibody reactive with *C. burnetii* antigen by ELISA, dot-ELISA, or latex agglutination. Confirmed cases of acute Q fever are those laboratory confirmations with clinical evidence of infection or an epidemiological link to a laboratory confirmation. Probable acute Q fever cases are with clinical evidence of infection and laboratory-supportive results. However, the non-specific clinical features and vague epidemiological clues of acute Q fever infection have increased the difficulty of diagnosis. For *C. burnetii* is an obligate intracellular gram-negative bacterium, it can't be cultured in standard laboratory (10). Up to now, serology is still the first-line method for testing *C. burnetii*. However, negative serology evidence shouldn't exclude the diagnosis. Melenotte et al. (10) and Imbert et al. (11) reported cases of Q fever with a negative or low antibody titer since IgM or IgG antibodies usually begin to appear until 2–4 weeks after the onset of the clinical symptoms. Moreover, serological methods are limited by the ways for obtaining antigens, the inconsistent analysis standards and expensive fluorescence microscope, thus hindering its widespread use, especially in non-epidemic areas (12). Molecular detection, such as PCR, can be positive when the acute infection is suspected before the antibody response (13–15). But targeted methods could be carried out only when clinicians suspect Q fever in acutely febrile patients. Therefore, the diagnosis of Q fever poses a challenge, especially at the early stage of infection.

Recently, mNGS detection, a high-throughput sequencing technique, has become a promising way to detect nucleic acid sequences from DNA/RNA of unknown origin directly, overcoming many of the limitations of conventional microbiological detection such as culture and serology methods (16). It has a great advantage in the diagnosis of complex and severe infections, such as respiratory tract infection (17, 18), joint infection (19), encephalitis (20), and microbial keratitis and endophthalmitis (21, 22). As a novel pathogen identification method, mNGS also can make a fast detection of difficult-to-culture pathogens such as *C. burnetii* (23, 24). Mingxing Huang reported the epidemic of acute Q fever in Zhuhai city confirmed by mNGS, which shows mNGS has higher sensitivity compared to 72.2% seropositivity rate and 95% qPCR positive rate (25). Owing to the complexity of mNGS results, interpreting them may be the greatest challenge. Clinical relevance and efficacy evaluation remain essential when interpreting results generated from mNGS. In our case, mNGS findings were correlated with clinical presentation on *C. burnetii* and antibiotic treatment with doxycycline is effective. Therefore, our case offers further proof that mNGS paves the way for the diagnosis of Q fever. This may solve the low sensitivity and specificity of diagnosis of *C. burnetii* infection, but it still needs clinical evaluation.

Neurological complications in acute Q fever including aseptic meningitis and meningoencephalitis are rarely reported (1, 26, 27). But Kofteridis et al. (28) reported that the clinical evidence of CNS involvement is not a rare feature of acute Q fever infection and *C. burnetii* should be considered as a possible etiology of meningitis or meningoencephalitis in endemic areas. In our study, this patient exhibited fever, severe headache, positive meningeal irritation signs and a mildly elevated white blood cell (count with a lymphocytic predominance) in CSF. All of those evidence did support the diagnosis of meningitis. Our patient's condition dramatically improved after doxycycline treatment, similar to other clinical cases described by Kofteridis which seem to be benign with a favorable outcome. Among meningitis due to *C. burnetii*, there is a lymphocytic predominance with proteins or glucose nearly normal in the CSF, consistent with my CSF changes (29, 30). Identification of *C. burnetii* in CSF samples with Q fever by PCR (31), it was suggested that *C. burnetii* may directly damage infected CNS tissue. Therefore, acute Q fever should be considered as a cause of acute meningitis. However, in our case, mNGS identified *C. burnetii* in patient blood, but not CSF. Former studies indicated that neurological complications might be related to circulating immune complexes (32). We thought it was also the possible pathogenesis in our case. Since aseptic meningitis may be a direct infection of *C. burnetii* to CNS or inflammatory reactions to systemic infection, we recommend detecting mNGS both in blood and CSF.

Although Q fever infections are not rare in China, clinical cases are still largely underdiagnosed on account of insufficient awareness and lacking epidemiological evidence. Furthermore, most of the published case reports in our country are lacking typical clinical presentations. If not treated timely, about 10–20% had neurological sequelae (27, 33) and about 1% of acute Q fever patients have been reported to be developed into the chronic form presenting with endocarditis and endovascular (34). Due to high pathogenicity and strong resistance, it is particularly important to make early diagnosis and effective antibiotic treatment. We reported an acute Q fever case with rare neurological complications (aseptic meningitis) which was quickly diagnosed by mNGS in Hunan.

## Data Availability Statement

The datasets used and/or analyzed during the current study are available from the corresponding author on reasonable request.

## Ethics Statement

Written informed consent was obtained from the individual(s) for the publication of any potentially identifiable images or data included in this article.

## Author Contributions

MG collected the materials, analyzed the data, and drafted the manuscript. XM and ZT assisted in clinical follow up. WW assessed the patient, performed the review of the literature, and edited the manuscript and tables for important intellectual content. JT provided his experience and edited the manuscript for important intellectual content. All authors contributed to the article and approved the submitted version.

## Funding

This work was supported by the Program of National Natural Science Foundation of China (Grant No. 81601083).

## Conflict of Interest

The authors declare that the research was conducted in the absence of any commercial or financial relationships that could be construed as a potential conflict of interest.

## Publisher's Note

All claims expressed in this article are solely those of the authors and do not necessarily represent those of their affiliated organizations, or those of the publisher, the editors and the reviewers. Any product that may be evaluated in this article, or claim that may be made by its manufacturer, is not guaranteed or endorsed by the publisher.
